# Supplementary material for: Horizontal gene transfer is predicted to overcome the diversity limit of competing microbial species
Source: Nat Commun. 2024 Jan 27;15:800. doi: 10.1038/s41467-024-45154-w (PMC10821886; doi:10.1038/s41467-024-45154-w)
Supplement: Supplementary file 1 — Supplementary Information [file 41467_2024_45154_MOESM1_ESM.pdf]

Supplementary Information for

Horizontal gene transfer is predicted to overcome the diversity limit of competing microbial species

Shiben Zhu[1], Juken Hong[1], Teng Wang[1]✉

[1]Key Laboratory of Quantitative Synthetic Biology, Shenzhen Institute of Synthetic Biology, Shenzhen Institutes of Advanced Technology, Chinese Academy of Sciences, Shenzhen 518055, China.

✉Corresponding author. Email: t.wang1@siat.ac.cn.

Supplementary Text

1. Parameter units, model development and the effective range of η

To better explain the parameter units in our analysis, let's first consider a model that describes population abundances in $cells \cdot mL^{-1}$. For a community of two competing species, the model is composed of four ODEs:

$$\frac{dS_1}{dt} = \mu_1^e S_1 \left(1 - \frac{S_1 + \gamma_2 S_2}{N_m}\right) - DS_1,$$

$$\frac{dS_2}{dt} = \mu_2^e S_2 \left(1 - \frac{S_2 + \gamma_1 S_1}{N_m}\right) - DS_2,$$

$$\frac{dP_1}{dt} = \mu_1(1 + \lambda_2)P_1 \left(1 - \frac{S_1 + \gamma_2 S_2}{N_m}\right) + \eta_1^c(S_2 + P_1)(S_1 - P_1) - (D + \kappa_1)P_1,$$

$$\frac{dp_2}{dt} = \mu_2(1 + \lambda_1)P_2 \left(1 - \frac{S_1 + \gamma_2 S_2}{N_m}\right) + \eta_2^c(S_1 + P_2)(S_2 - P_2) - (D + \kappa_2)P_2.$$

The units of the variables and parameters are:

variables and parameters	units
$S_1,\ S_2$	$cells \cdot mL^{-1}$
$P_1,\ P_2$	$cells \cdot mL^{-1}$
N_m	$cells \cdot mL^{-1}$
t	$hour$
$\mu_1^e,\ \mu_2^e,\ \mu_1,\ \mu_2$	$hour^{-1}$
$\gamma_1,\ \gamma_2$	1
D	$hour^{-1}$
$\kappa_1,\ \kappa_2$	$hour^{-1}$
$\lambda_1,\ \lambda_2$	1
$\eta_1^c,\ \eta_2^c$	$cells^{-1} \cdot mL \cdot hour^{-1}$

Here, N_m is maximum carrying capacity of the population. S_i and P_i $(i = 1, 2)$ represent the number of cells per unit of volume. γ_i and λ_i are dimensionless. η_i^c is the MGE transfer rate in the unit of $cells^{-1} \cdot mL \cdot hour^{-1}$. This unit was commonly used in empirical measurements of plasmid conjugation efficiencies[1].

Letting $s_i = S_i/N_m$, $p_i = P_i/N_m$ and $\eta_i = \eta_i^c N_m$, the model can be simplified into

$$\frac{ds_1}{dt} = \mu_1^e s_1(1 - s_1 - \gamma_2 s_2) - Ds_1,$$

$$\frac{ds_2}{dt} = \mu_2^e s_2(1 - s_2 - \gamma_1 s_1) - Ds_2,$$

$$\frac{dp_1}{dt} = \mu_1(1+\lambda_2)p_1(1-s_1-\gamma_2 s_2) + \eta_1(s_2+p_1)(s_1-p_1) - (D+\kappa_1)p_1,$$

$$\frac{dp_2}{dt} = \mu_2(1+\lambda_1)p_2(1-s_2-\gamma_1 s_1) + \eta_2(s_1+p_2)(s_2-p_2) - (D+\kappa_2)p_2.$$

This is the model version that we used in our analysis. We chose this version because it requires fewer parameters. s_i and p_i are dimensionless and represent the population abundances relative to the maximum carrying capacity. The units of s_i, p_i and η_i are:

variables and parameters	units
s_1, s_2	1
p_1, p_2	1
η_1, η_2	$hour^{-1}$

Since η_i is calculated by multiplying η_i^c (the empirical ones) by N_m, η_1 and η_2 values in our model are several orders of magnitude higher than η_1^c and η_2^c.

In this work, we explored the effects of HGT rate in the range of $0 < \eta_i < 0.5\ h^{-1}$. A previous study estimated the transfer rates of a conjugative plasmid cross species (from *Klebsiella pneumoniae* to *Escherichia coli*) and within species (between *E. coli* strains) to be around 10^{-13} and 10^{-7} $cells^{-1} \cdot mL \cdot hour^{-1}$, respectively[1]. Whether HGT rates in the empirically estimated range is sufficient to enable species coexistence is dependent on N_m, the maximum carrying capacity of the population. For instance, in colon where the bacterial density reaches 10^{12} cells per gram, η_i^c values as low as 10^{-13} can sufficiently promote the coexistence feasibility[2]. However, if the microbial density is low, the effects of HGT on species diversity might become negligible compared with other mechanisms such as growth trade-offs or cross-feeding[3,4]. We explicitly simulated the relationship between coexistence feasibility and η_i^c values under different values of N_m. Our results suggested that with high N_m, the empirically estimated range of η_i^c can generate non-negligible influences on coexistence (Supplementary Fig. 4a-c).

We also explored the influences of MGE fitness on the effective range of HGT rate. Our numerical simulations suggest that when MGEs encode growth benefits, very small transfer rates can lead to significant promotion of coexistence feasibility (Supplementary Fig. 4d). In contrast, when MGEs encode a mixture of burden and benefits, transfer rates need to be large enough to enable coexistence. These results predict that the contribution of HGT in natural environments is determined by the population density as well as the MGE fitness effects. The role of HGT tends to become more prominent where the maximum carrying capacity is high or when MGEs encode fitness benefits.

2. Epistasis of mobile genetic elements

In the two-species model, we assumed that the metabolic burden or benefit of an MGE was independent of the host species or strains. However, in nature the same MGE can have different fitness effects in different genetic backgrounds due to epistasis[5-8]. To evaluate the influence of this assumption on the conclusion, we built a model that accounted for epistasis. Specifically, for two competing species, we dissected their growth rates μ_1 and μ_2 into two components: the basal growth rate (μ_1^0 and μ_2^0), and the fitness effects (λ_{11} and λ_{22}) of the mobilizable genes. HGT creates a subpopulation (denoted as p_1 and p_2) within each species that acquires the mobilizable genes from its competitor. The

growth rates of p_1 and p_2 can be obtained as $\mu_1(1+\lambda_{12})$ and $\mu_2(1+\lambda_{21})$, respectively. Here, λ_{ij} $(i,j=1,2)$ describes the fitness effects of the j-th mobile gene in the i-th species. Without epistasis, the fitness effects are independent of the host species, which means $\lambda_{11}=\lambda_{21}$ and $\lambda_{22}=\lambda_{12}$. Epistasis leads to the difference between λ_{11} and λ_{21} (or between λ_{22} and λ_{12}). The population dynamics can be described by the following ODEs:

$$\frac{ds_1}{dt}=\mu_1(1+\lambda_{12}\frac{p_1}{s_1})s_1(1-s_1-\gamma_2 s_2)-Ds_1,$$

$$\frac{ds_2}{dt}=\mu_2(1+\lambda_{21}\frac{p_2}{s_2})s_2(1-s_2-\gamma_1 s_1)-Ds_2,$$

$$\frac{dp_1}{dt}=\mu_1(1+\lambda_{12})p_1(1-s_1-\gamma_2 s_2)+\eta_1(s_2+p_1)(s_1-p_1)-(D+\kappa_1)p_1,$$

$$\frac{dp_2}{dt}=\mu_2(1+\lambda_{21})p_2(1-s_2-\gamma_1 s_1)+\eta_2(s_1+p_2)(s_2-p_2)-(D+\kappa_2)p_2.$$

Here, γ_1 and γ_2 are the competition strengths. D is the dilution rate. η_1 and η_2 are transfer rates. κ_1 and κ_2 are the loss rates of the mobilizable genes.

The epistasis can be quantitatively described by two ratios: $\xi_1=\lambda_{21}/\lambda_{11}$ and $\xi_2=\lambda_{12}/\lambda_{22}$. $\xi_1>0$ and $\xi_2>0$ represent magnitude epistasis, where the host genomic background only influences the magnitude but not the sign of the fitness effect. In contrast, $\xi_1<0$ or $\xi_2<0$ represent the sign epistasis, where the same MGE causes growth burden in one species while brings fitness benefit in the other species. $\xi_1=1$ and $\xi_2=1$ represent no epistasis. In numerical simulations, we tested both types of epistasis. Specifically, we calculated the coexistence feasibility of the two species under different ξ_1 and ξ_2 values, by numerical simulations with randomized parameters. Given competition strength, we randomized μ_1 and μ_2 multiple times following uniform distributions while keeping μ_1^0 and μ_2^0 constants. Next, we simulated the population dynamics until steady states and calculated the feasibility as the fraction of growth rate combinations leading to coexistence. As shown in Supplementary Fig. 5, our numerical results suggested that how HGT affects coexistence is dependent on the epistasis type. Magnitude epistasis doesn't qualitatively change the conclusion but sign epistasis does. When a mobile gene causes opposite fitness effects on two different species, the transfer of this gene will reduce their coexistence feasibility. These results suggest that MGE epistasis might add another layer of complexities into the interplay between HGT and species coexistence.

3. The effect of HGT on species coexistence when mobile genes promote inter-species competition

Our main model assumed that the MGEs only affected species growth rates and the transfer of these genes wouldn't change the strength of inter-species competition. However, the sharing of many mobile genes can also promote niche overlapping, leading to the increase of competition strength[5,9]. To understand how the transfer of these genes will influence species coexistence, we adapted the main model, by considering the dynamic change of competition strength during gene transfer. For a population of two competing species, the model consists of four ODEs:

$$\frac{ds_1}{dt}=\mu_1 s_1[1-s_1-(\gamma_2+\delta_2\frac{p_2}{s_2})s_2]-Ds_1,$$

$$\frac{ds_2}{dt}=\mu_2 s_2[1-s_2-(\gamma_1+\delta_1\frac{p_1}{s_1})s_1]-Ds_2,$$

$$\frac{dp_1}{dt} = \mu_1 p_1[1 - s_1 - (\gamma_2 + \delta_2 \frac{p_2}{s_2}) s_2] + \eta_1(s_2 + p_1)(s_1 - p_1) - (D + \kappa_1) p_1,$$

$$\frac{dp_2}{dt} = \mu_2 p_2[1 - s_2 - (\gamma_1 + \delta_1 \frac{p_1}{s_1}) s_1] + \eta_2(s_1 + p_2)(s_2 - p_2) - (D + \kappa_2) p_2.$$

μ_1 and μ_2 are the maximum growth rates of the two species, which are independent of the gene transfer process. Here, we assumed that mobile genes didn't cause fitness burden or benefits on the species growth rates. D is the dilution rate. η_1 and η_2 are transfer rates. κ_1 and κ_2 are the loss rates of the mobilizable genes. The interspecies competitions are determined by two components: the basal competition strengths (γ_1 and γ_2) and the added parts by HGT (δ_1 and δ_2). The overall competition strength is calculated as $\gamma_1 + \delta_1 \frac{p_1}{s_1}$ and $\gamma_2 + \delta_2 \frac{p_2}{s_2}$, respectively. Positive δ_1 and δ_2 values represent HGT promoting the inter-species competition.

This framework can be readily extended to complex communities of multiple species. Consider a community of m species. The population dynamics can be described by $2m$ ODEs as follows:

$$\frac{ds_i}{dt} = \mu_i s_i [1 - s_i - \sum_{j \neq i} (\gamma_{ji} + \delta_{ji} \frac{p_{ji}}{s_j}) s_j] - D s_i,$$

$$\frac{dp_{ij}}{dt} = \mu_i p_{ij} [1 - s_i - \sum_{j \neq i} (\gamma_{ji} + \delta_{ji} \frac{p_{ji}}{s_j}) s_j] + (s_i - p_{ij}) \sum_{k=1}^{m} \eta_{jki} p_{kj} - (D + \kappa_{ij}) p_{ij}. \quad (i \neq j).$$

s_i represents the abundance of the i-th species, and p_{ij} represents the abundance of cells in the i-th species that acquires s_j-originated mobile genes. We assumed $p_{ii} = s_i$. μ_i is the maximum growth rate of s_i. Here, we ignored the fitness effects of mobile genetic elements on species growth rates. γ_{ji} stands for the basal interaction that s_j imposes on the i-th species without HGT. δ_{ji} describes the change of inter-species competition due to gene transfer. The overall interaction that s_j imposes on the i-th species becomes $\gamma_{ji} + \delta_{ji} \frac{p_{ji}}{s_j}$. η_{jki} is the transfer rate of the s_j-originated genes from species k to species i. D and κ_{ij} are the dilution and gene loss rate, respectively.

For a community of m species, we calculated the coexistence feasibility under different δ_{ji} values to analyze the influence of HGT on species coexistence. Specifically, we randomized μ_{ij} values multiple times following uniform distributions. Next, we simulated the population dynamics until steady states and calculated the feasibility as the fraction of growth rate combinations leading to coexistence. As shown in Supplementary Fig. 6, with δ_{ji}=0, which means mobile genes have no effects on inter-species competition, increasing HGT rate will not affect species coexistence. However, when δ_{ji} is positive, increasing gene transfer rate reduces the coexistence feasibility, regardless of species number in the community. These results suggest that when mobile genes promote inter-species competition, HGT can have negative impact on the diversity of competing species.

4. The effect of HGT on multi-stability of microbial communities

Multi-stable microbial communities have more than one stable states in the same conditions[10]. When a community is multi-stable, large perturbations in species abundances can drive the drastic regime shift of population compositions[10]. To understand how HGT influences the multi-stability of microbial communities, we considered a population of 8 competing microbes. The inter-species competition strength was set to be larger than 1, such that multiple stable states would exist. To approximate the number of alternative stable states, we randomized the initial abundance of each species for 500 times. For each randomized initial condition, we simulated the population dynamics till the system reached steady state. Each steady state can be described by a vector of species abundances $[s_1, s_2, \dots, s_8]$. Here s_i stands for the steady-state abundance of the i-th species. Then we clustered the 500 steady states into different attractors, based on the Euclidean distances among different steady states. Steady states with distance smaller than 0.05 were clustered into the same attractor. Our numerical results suggest that the number of alternative stable states increases with HGT rate, indicating the role of gene transfer on the global stability landscape of microbial communities (Supplementary Fig. 7).

5. Discrete fitness effect of MGEs

The fitness effect of MGEs can become discrete when the loss of the MGEs results in cell death. For instance, under strong antibiotic selections, only cells carrying the antibiotic resistant MGEs can survive. To examine whether our conclusion is still applicable in this scenario, we generalized our model by considering the transfer of an antibiotic resistant MGE in a population of m species. Within each species, only MGE-carrying cells can grow, with μ_i being their maximum growth rate. The MGE-free cells stop growing, and will be eventually depleted by dilution D. Therefore, the community dynamics can be described by $2m$ ODEs ($i = 1,2, \dots, m$):

$$\frac{ds_i}{dt} = \mu_i p_i \left(1 - s_i - \sum_{j \neq i} \gamma_{ji} s_j\right) - D s_i,$$

$$\frac{dp_i}{dt} = \mu_i p_i \left(1 - s_i - \sum_{j \neq i} \gamma_{ji} s_j\right) + (s_i - p_i) \sum_{j=1}^{m} \eta_{ji} p_j - \kappa_i p_i - D p_i.$$

s_i is the total abundance of the i-th species, and p_i is the abundance of cells carrying the MGE in s_i. In the first equation, the first term describes the overall growth of the i-th species, which is only contributed by p_i since the MGE-free cells ($s_i - p_i$) are not able to grow. η_{ji} is the MGE transfer rate from the j-th to the i-th species. κ_i is MGE loss rate in the i-th species. γ_{ji} describes the inter-species competition strength.

Without loss of generality, we assumed the species 1 to be the MGE donor and p_1 to be equal with s_1. Without HGT, the MGE is only present in the donor species, while HGT allows the MGE to spread to other species. To understand how HGT rate influences microbial diversity, we performed numerical simulations on communities of 2, 5 and 10 species. Specifically, we tested different transfer rates from 0 to 0.5 h^{-1}, and calculated the diversity of each population at steady states. Our numerical results suggest that increasing HGT rate promotes the species coexistence and diversity (Supplementary Fig. 9). This result can be intuitively understood as follows: without HGT, only the donor species carrying the MGE can survive due to the antibiotic selection, while HGT spread the MGE to other species, allowing more species to coexist with the donor species.

6. The diversity of MGEs

In our model, we assumed the number of the MGEs equaled the species number. To understand whether our conclusion is still applicable when the diversity of MGEs is higher than bacterial chromosomes, we established an extended model that accounts for the flow of an arbitrary number (denoted as n) of MGEs in a community of m species:

$$\frac{ds_i}{dt} = \mu_i^0 \alpha_i s_i \left(1 - s_i - \sum_{j \neq i} \gamma_{ji} s_j \right) - D s_i,$$

$$\frac{dp_{ij}}{dt} = \mu_i^0 (1 + \lambda_{ij}) \beta_{ij} p_{ij} \left(1 - s_i - \sum_{j \neq i} \gamma_{ji} s_j \right) + \left(s_i - p_{ij} \right) \sum_{k=1}^{m} \eta_{jki} p_{kj} - \kappa_{ij} p_{ij} - D p_{ij}.$$

Here, s_i $(i = 1,2, \dots, m)$ is the abundance of the i-th species. p_{ij} $(i = 1,2, \dots, m; j = 1,2, \dots, n)$ is the abundance of the cells that carry the j-th MGE in species i. μ_i^0 is the basal growth rate of s_i determined by non-mobilizable genes. α_i describes the overall fitness effect of all the MGEs that the i-th species carries: $\alpha_i = \prod_{j=1}^{n} (1 + \lambda_{ij} \frac{p_{ij}}{s_i})$. λ_{ij} is the fitness effect of the j-th MGE in the i-th species. γ_{ji} represents the interaction strength that the j-th species imposes on the i-th species. β_{ij} is the overall fitness effect of all the other MGEs: $\beta_{ij} = \prod_{k \neq j} (1 + \lambda_{ik} \frac{p_{ik}}{s_i})$. The second term in the second equation describes the MGE transfer from donors (p_{kj}) to recipients ($s_i - p_{ij}$), where η_{jki} represents the transfer rate of the j-th MGE from species k to species i. κ_{ij} is the loss rate of the j-th MGE in the i-the species.

When the number of MGEs is larger than species number, some species will carry more than one MGEs. $M(j)$ represents the mapping from a MGE to its initial host species. For instance, $M(2) = 3$ represents species 3 being the initial host of the second MGE. We randomized the values of $M(j)$ between 1 and m to ensure that each MGE is initially carried by a single species. In numerical simulations, we considered communities of 2, 3, 5 or 8 species. For each species number, we tested three different ratios between MGE number and species number. The growth rates of different species were randomized between 0.25 and 0.75 h^{-1}. Then we calculated the steady-state species number by applying a cut-off value 0.01. A species with the abundance lower than this cut-off value was treated as out-competed. Next, we repeated the simulations for 500 times with randomized growth rates and calculated the coexistence feasibility. Our results suggest that increasing HGT rate promotes the coexistence feasibility, regardless of the MGE diversities (Supplementary Fig. 10).

Supplementary Figures

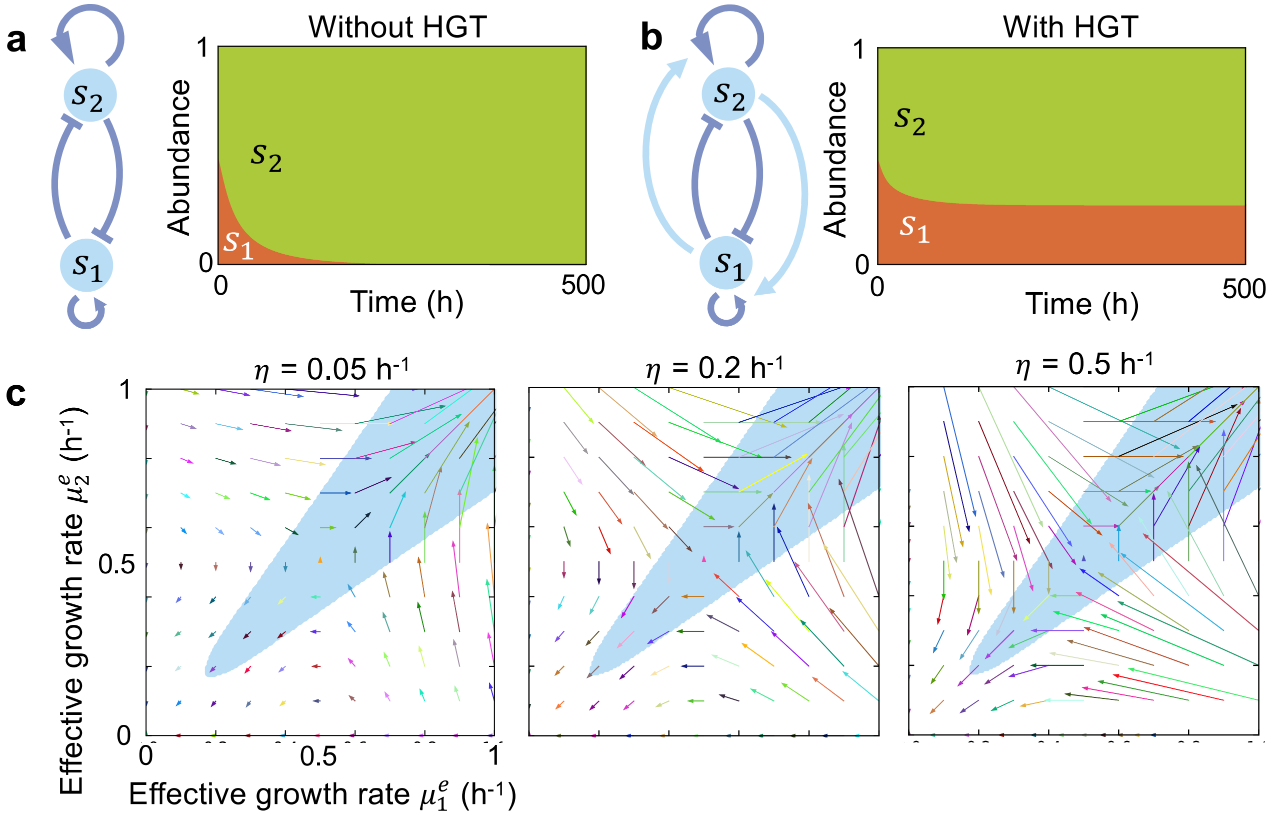

Supplementary Fig. 1| HGT enables the coexistence of two competing species which would otherwise outcompete each other. a and b Examples of population dynamics with (right) or without (left) HGT. Numerical simulations were performed with $\mu_1^0 = \mu_2^0 = 0.5\, h^{-1}$, $\lambda_1 = -0.2$, $\lambda_2 = 0.2$, $\gamma_1 = \gamma_2 = 0.8$, $D = 0.2\, h^{-1}$, $\kappa = 0.05\, h^{-1}$. Without HGT ($\eta = 0$), species 1 is completely outcompeted by species 2; while with HGT ($\eta = 0.2\, h^{-1}$), the two species coexist stably. **c** Gene transfer leads to the dynamic change of the effective growth rates of the two species. The shaded area represents the parameter region that leads to coexistence. Each arrow links the static growth rates without HGT and the effective growth rates after 20 hours with HGT. The parameters used in the simulations are $\mu_1^0 = \mu_2^0 = 0.5\, h^{-1}$, $\gamma_1 = \gamma_2 = 0.99$, $D = 0.2\, h^{-1}$, $\kappa = 0.05\, h^{-1}$. Three HGT rates were tested from left to right.

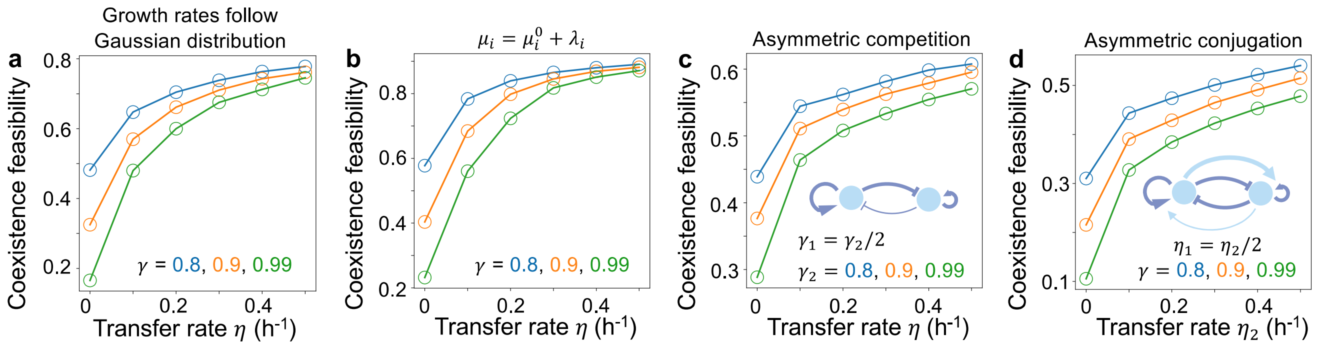

Supplementary Fig. 2| HGT promotes the coexistence of two competing species regardless of a variety of confounding factors. a Coexistence feasibility increases with η when μ_1 and μ_2 follow Gaussian distribution with mean being 0.5 h^{-1} and standard deviation being 0.2 h^{-1}. Three different values for γ_1 and γ_2 (marked with different colors) were tested. **b** Coexistence feasibility increases with η when the growth rates were calculated by adding μ_i^0 and λ_i. λ_1 and λ_2 were randomized (n = 2000 replicates) between -0.25 h^{-1} and 0.25 h^{-1} following uniform distributions while μ_1^0 and μ_2^0 were kept as constants. **c** HGT promotes the coexistence of two species when their interspecies competitions are asymmetric. γ_1 or γ_2 represents the negative interaction that species 1 or 2 imposes on its counterpart, respectively. Here, we assumed $\gamma_1 = \gamma_2/2$ and tested three different γ_2 values. **d** HGT promotes the coexistence of two species when their interspecies gene transfer rates are asymmetric. η_1 or η_2 represents the gene transfer rate from species 1 or 2 to its counterpart, respectively. Here, we assumed $\eta_1 = \eta_2/2$. Three different values for γ_1 and γ_2 (marked with different colors) were tested.

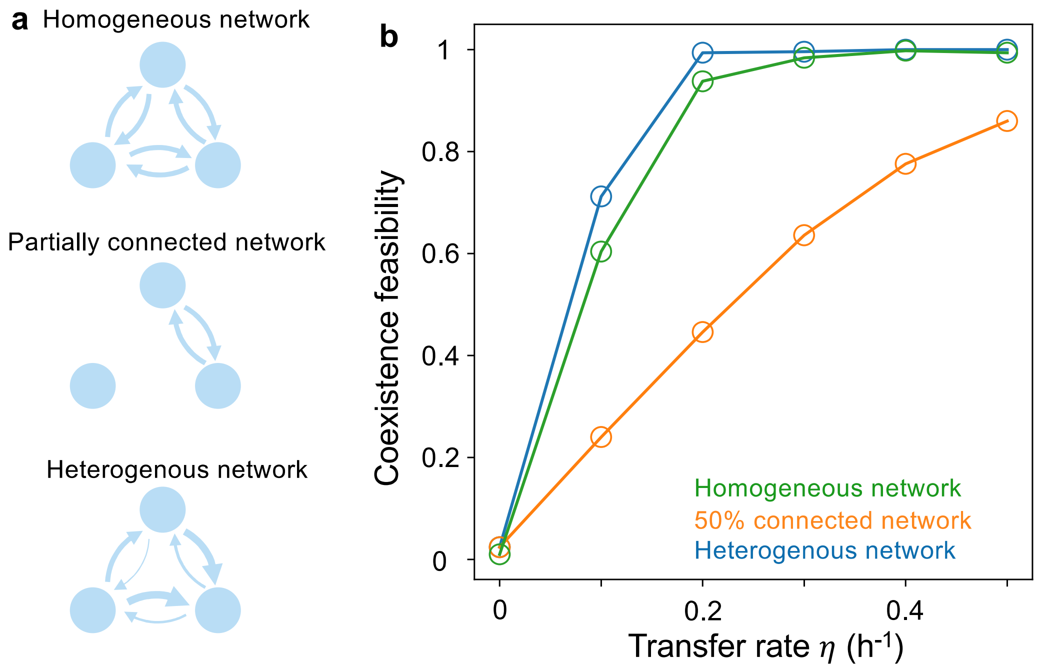

Supplementary Fig. 3| HGT promotes the coexistence feasibility of multiple competing species regardless of the architecture of gene transfer network. a Schematics of different network types. Filled circles stand for different species. Directed arrows represent gene transfer from donors to recipients, and the arrow width represents the transfer rate. Population of three species are shown as examples here. In homogeneous networks, each species transfers the genes with the same rate. In partially connected networks, the gene is only able to be transferred between a fraction of species pairs. Heterogeneous networks are fully connected, with large variations of transfer rates. **b** The effect of HGT does not rely on the architecture of gene transfer network. The coexistence feasibility of five competing species was calculated by randomizing growth rates between 0.4 and 0.6 h^{-1} following uniform distributions (n = 500 replicates). 50% connectance was used as an example of partially connected networks. In heterogenous networks, the gene transfer rates were randomized between 0 and 2η following uniform distributions. Other parameters are $\gamma_{ij} = 0.9$, $\kappa = 0.005\ h^{-1}$, $D = 0.2\ h^{-1}$.

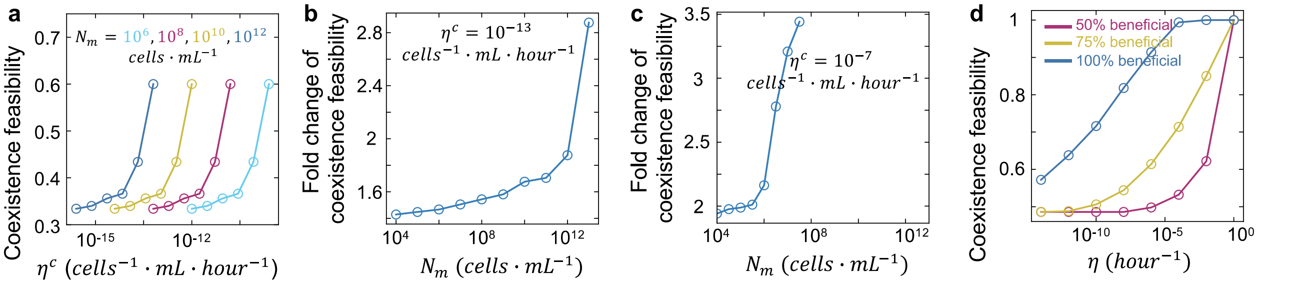

Supplementary Fig. 4| The effective range of HGT rate is determined by maximum carrying capacity and MGE fitness effect. a The relationship between coexistence feasibility of two competing species and η^c under different N_m values. η^c has the unit of $cells^{-1} \cdot mL \cdot hour^{-1}$, corresponding to the empirically measured gene transfer rates. η^c needs to be multiplied by the maximum carrying capacity N_m before being plugged into our model. In other words, η in our model equals $\eta^c \cdot N_m$. When N_m is large, even small η^c can lead to large increase of coexistence feasibility. Here, coexistence feasibilities were calculated by randomizing μ_1 and μ_2 between 0 and 1 h^{-1} following uniform distributions (n = 500 replicates). Other parameters are $\gamma = 0.9$, $\kappa = 0.005\, h^{-1}$, $D = 0.2\, h^{-1}$. **b-c** The effectiveness of HGT on promoting species coexistence increases with N_m. Two different η^c values, 10^{-13} and $10^{-7}\, cells^{-1} \cdot mL \cdot hour^{-1}$ were tested. The HGT effectiveness was calculated by dividing the coexistence feasibility with HGT by the feasibility without HGT. **d** Small HGT rate is sufficient to promote coexistence feasibility when MGEs are beneficial. When calculating coexistence feasibility, we randomized the fitness effects of each MGE in certain ranges. Here, we considered three different scenarios: MGEs are 50%, 75% or 100% likely to be beneficial. The results are shown in three different colors.

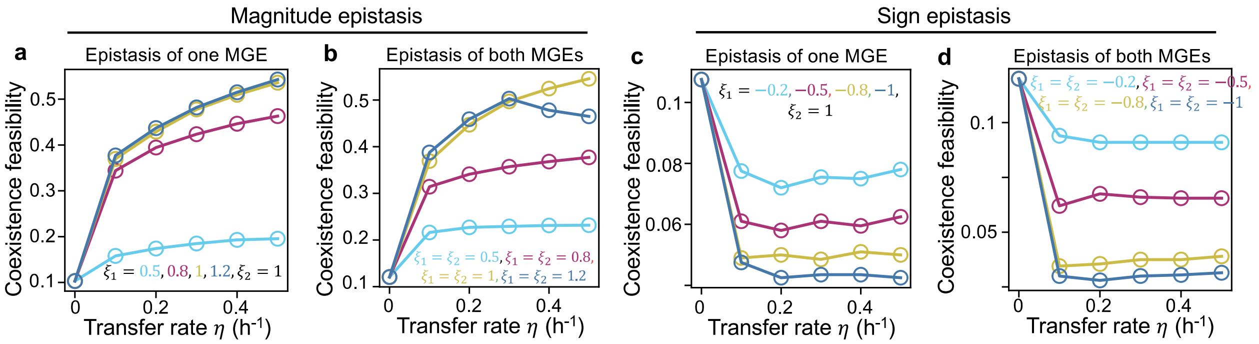

Supplementary Fig. 5| The influence of epistasis on the interplay between HGT and species coexistence. a-b With magnitude epistasis, increasing HGT rate still promotes the coexistence feasibility of two competing species. **c-d** With sign epistasis, where the same MGE causes growth burden in one species while brings fitness benefit in the other, increasing HGT rate reduces the coexistence feasibility. For each type of epistasis, we considered two scenarios. In a and c, host genetic background influences the fitness effect of only one MGE, while in b and d, host genetic background influences both MGEs. ξ_1 and ξ_2 are defined as $\lambda_{21}/\lambda_{11}$ and $\lambda_{12}/\lambda_{22}$, respectively. $\xi_1 > 0$ and $\xi_2 > 0$ represent magnitude epistasis, while $\xi_1 < 0$ or $\xi_2 < 0$ represents the sign epistasis. $\xi_1 = 1$ and $\xi_2 = 1$ represent no epistasis. For each η, we calculated the coexistence feasibility by randomizing μ_1 and μ_2 between 0 and 1 h^{-1} (n = 2000 replicates) following uniform distributions. Other parameters are $\gamma_1 = \gamma_2 = 0.99$, $\kappa = 0.005\ h^{-1}$, $D = 0.2\ h^{-1}$.

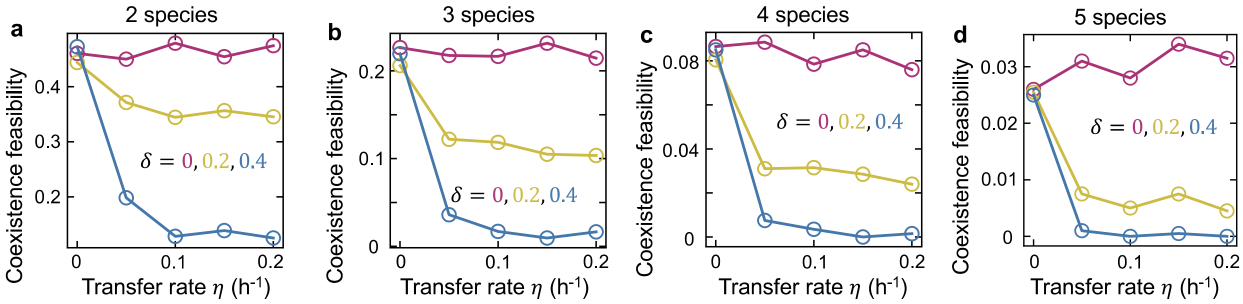

Supplementary Fig. 6| When mobile genes promote inter-specific competition, HGT reduces the coexistence feasibility of competing species. **a** Coexistence feasibility decreases with HGT rate in communities of 2 species. **b** Coexistence feasibility decreases with HGT rate in communities of 3 species. **c** Coexistence feasibility decreases with HGT rate in communities of 4 species. **d** Coexistence feasibility decreases with HGT rate in communities of 5 species. Here, δ describes the effect of mobile genes on the competition strength. Positive δ represents HGT promoting competition. In numerical simulations, we tested three different δ values. When calculating the coexistence feasibility, we randomized all the μ_{ij} values between 0 and 1 h^{-1} following uniform distributions (n = 2000 replicates). Other parameters are $\gamma_{ij} = 0.6$, $\kappa = 0.005\ h^{-1}$, $D = 0.2\ h^{-1}$.

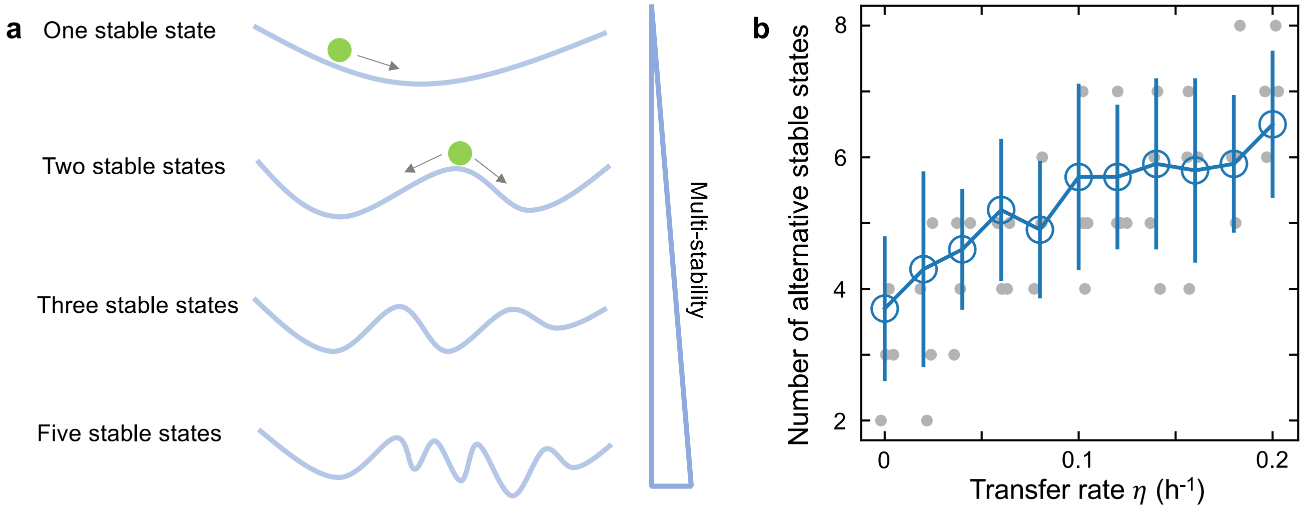

Supplementary Fig. 7| HGT promotes the emergence of alternative stable states in microbial communities. a A Schematic of the alternative stable states in microbial populations. When multiple stable states exist, the steady-state composition of the population depends on the initial abundances of difference species. **b** The number of alternative stable states increases with HGT rate. We considered populations of 8 competing species. The strength of interspecies competition equals 1.1. μ_0 equals 0.5 h^{-1}, while λ_{ij} were randomized between -0.4 and 0.4 following uniform distributions. Other parameters are $D = 0.2\ h^{-1}$, $\kappa = 0.005\ h^{-1}$. For each parameter setting, we randomized the initial abundances of different species for 500 times. For each initial condition, we simulated the population dynamics till the system reached the steady state. Then we calculated the number of stable states based on the steady-state species abundances in the 500 simulations. The data are presented as mean $\pm$ standard deviation of 10 independent replicates.

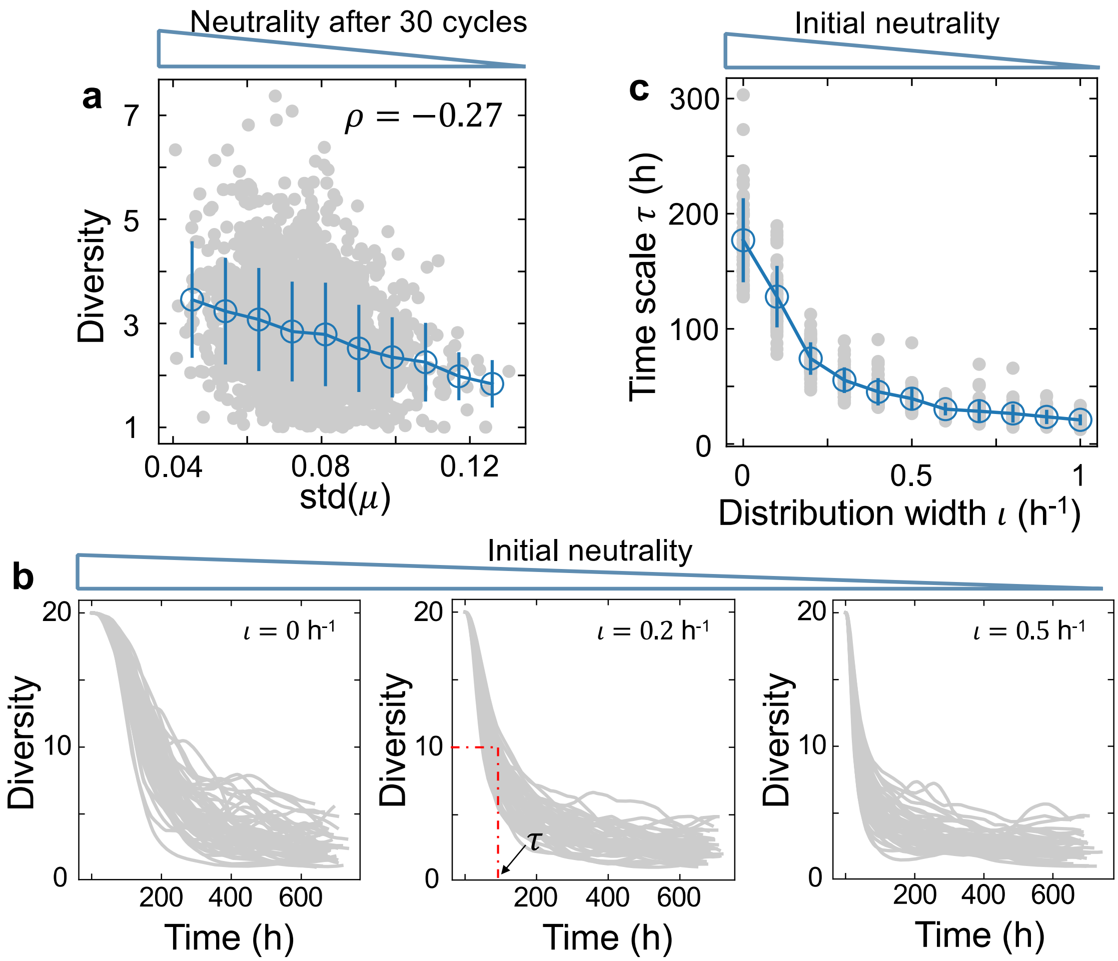

Supplementary Fig. 8| Neutrality determines the robustness of community diversity to fitness perturbations. a The relationship between species diversity and the standard deviation of growth rates after 30 cycles of random perturbations without HGT. Here, we considered communities of 20 competing species. 2000 repeated simulations were initiated with perfect neutrality, where all species had the same growth rates $\mu_i = 0.5\, h^{-1}$ and identical abundances. Perturbations were introduced into growth rates at random intervals. Each perturbation caused the random variations of growth rates with the magnitude of less than 5%. Other parameters are $D = 0.2\, h^{-1}$, $\gamma_{ij} = 0.9$, $\kappa = 0.005\, h^{-1}$. ρ is Spearman correlation coefficient between $std(\mu)$ and diversity (p value = 0). The x axis was divided into 10 bins with widths of 0.0088. The bar plots represent the mean values +/− standard deviations of all the diversity values within each bin. Each bin contains 8 to 493 independent replicates. **b** The simulated dynamics of communities composed of 20 competing species without HGT. At time zero, the growth rates of different species were randomized around 0.5 h^{-1} following uniform distributions. l represents the width of the distribution. Smaller ι stands for greater initial neutrality. After time zero, perturbations were introduced into species growth rates at random intervals. For each simulation, we calculated the time value τ when the population diversity decreased from 20 to 10. τ characterizes how quickly the system will reach a steady state. Here, we tested three different ι values, and for each ι, we repeated the simulation 50 times. **c** The relationship between distribution width ι and time scale τ. Greater l, corresponding weaker initial neutrality, leads to faster decrease of community diversity under random perturbations. Data are represented as mean $\pm$ standard deviation of 50 replicates.

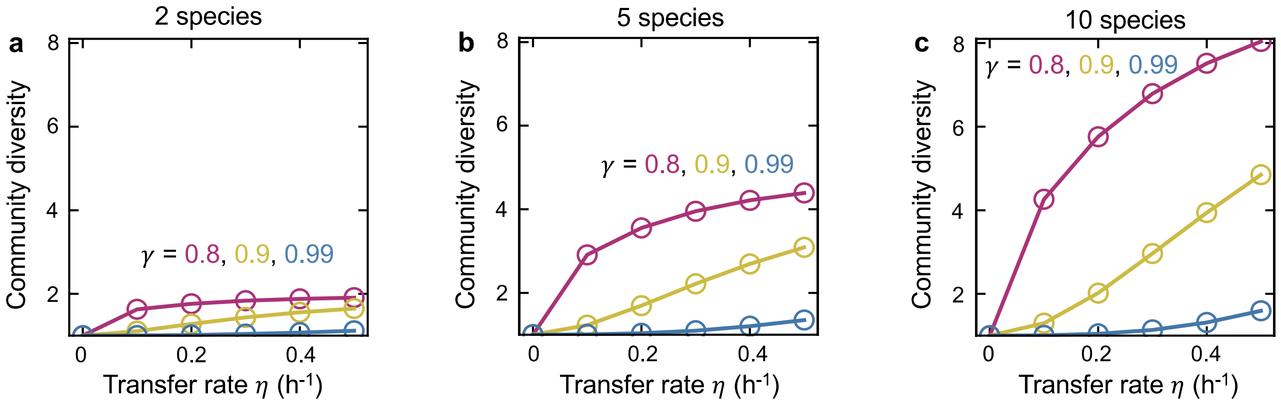

Supplementary Fig. 9| HGT promotes the species diversity when the fitness effect of the MGE is discrete. a Species diversity increases with HGT rate in communities of 2 species. **b** Species diversity increases with HGT rate in communities of 5 species. **c** Species diversity increases with HGT rate in communities of 10 species. Here, we considered the scenario where the MGE encoded resistance to an antibiotic. Under strong antibiotic selection, only cells carrying the MGE can survive. For each population, three different inter-species competition strengths were tested. The community diversity was calculated as $e^{-\sum \frac{s_i}{s_T} log \frac{s_i}{s_T}}$, where s_i is the abundance of the i-th species and s_T is the total abundance of all species. Other parameters are $\mu_i = 0.5\ h^{-1}$, $\kappa_i = 0.05\ h^{-1}$, $D = 0.2\ h^{-1}$.

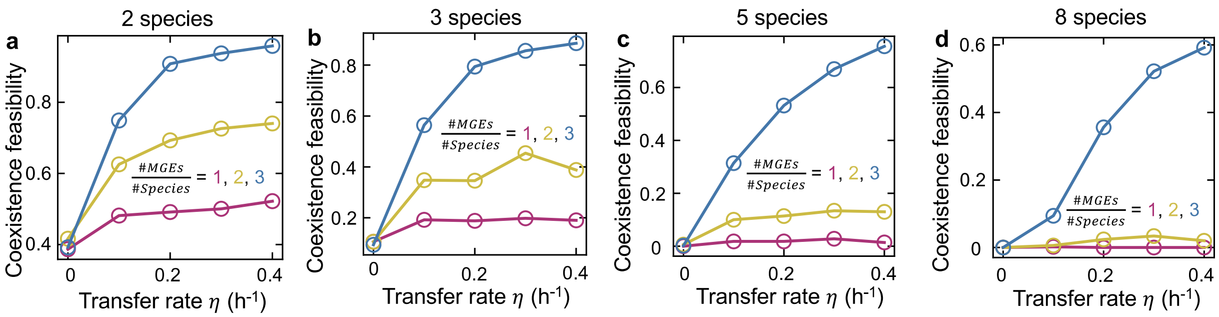

Supplementary Fig. 10| Increasing HGT rate promotes the coexistence feasibility regardless of MGE diversities. a Coexistence feasibility increases with HGT rate in communities of 2 species. **b** Coexistence feasibility increases with HGT rate in communities of 3 species. **c** Coexistence feasibility increases with HGT rate in communities of 5 species. **d** Coexistence feasibility increases with HGT rate in communities of 8 species. For each community, we tested three different ratios between MGE number and species number (marked by different colors). Coexistence feasibilities were calculated by randomizing species growth rate (n = 500 replicates) between 0.25 and 0.75 h^{-1} following uniform distributions. Other parameters were $\gamma_{ij} = 0.9$, $\kappa_{ij} = 0.005\ h^{-1}$, $D = 0.2\ h^{-1}$.

References

1. Kosterlitz, O. *et al.* Estimating the transfer rates of bacterial plasmids with an adapted Luria–Delbrück fluctuation analysis. *PLoS Biology* **20**, e3001732 (2022).
2. Dieterich, W., Schink, M. & Zopf, Y. Microbiota in the gastrointestinal tract. *Medical Sciences* **6**, 116 (2018).
3. Manhart, M. & Shakhnovich, E. I. Growth tradeoffs produce complex microbial communities on a single limiting resource. *Nature Communications* **9**, 3214 (2018).
4. Goldford, J. E. *et al.* Emergent simplicity in microbial community assembly. *Science* **361**, 469-474 (2018).
5. Acar Kirit, H., Lagator, M. & Bollback, J. P. Experimental determination of evolutionary barriers to horizontal gene transfer. *BMC Microbiology* **20**, 1-13 (2020).
6. Gama, J. A., Zilhão, R. & Dionisio, F. Plasmid interactions can improve plasmid persistence in bacterial populations. *Frontiers in Microbiology* **11**, 2033 (2020).
7. Silva, R. F. *et al.* Pervasive sign epistasis between conjugative plasmids and drug-resistance chromosomal mutations. *PLoS Genetics* **7**, e1002181 (2011).
8. San Millan, A., Heilbron, K. & MacLean, R. C. Positive epistasis between co-infecting plasmids promotes plasmid survival in bacterial populations. *The ISME Journal* **8**, 601-612 (2014).
9. Bonham, K. S., Wolfe, B. E. & Dutton, R. J. Extensive horizontal gene transfer in cheese-associated bacteria. *Elife* **6**, e22144 (2017).
10. Gonze, D., Lahti, L., Raes, J. & Faust, K. Multi-stability and the origin of microbial community types. *The ISME Journal* **11**, 2159-2166 (2017).
