# Supplementary material for: Horizontal gene transfer is predicted to overcome the diversity limit of competing microbial species
Source: Nat Commun. 2024 Jan 27;15:800. doi: 10.1038/s41467-024-45154-w (PMC10821886; doi:10.1038/s41467-024-45154-w)
Supplement: Supplementary file 3 — Reporting Summary [file 41467_2024_45154_MOESM3_ESM.pdf]

nature portfolio

Corresponding author(s): Teng Wang

Last updated by author(s): 2023/12/28

# Reporting Summary

Nature Portfolio wishes to improve the reproducibility of the work that we publish. This form provides structure for consistency and transparency in reporting. For further information on Nature Portfolio policies, see our Editorial Policies and the Editorial Policy Checklist.

## Statistics

For all statistical analyses, confirm that the following items are present in the figure legend, table legend, main text, or Methods section.

| n/a | Confirmed | |
|---|---|---|
| ☐ | ☒ | The exact sample size (*n*) for each experimental group/condition, given as a discrete number and unit of measurement |
| ☐ | ☒ | A statement on whether measurements were taken from distinct samples or whether the same sample was measured repeatedly |
| ☒ | ☐ | The statistical test(s) used AND whether they are one- or two-sided<br>*Only common tests should be described solely by name; describe more complex techniques in the Methods section.* |
| ☒ | ☐ | A description of all covariates tested |
| ☐ | ☒ | A description of any assumptions or corrections, such as tests of normality and adjustment for multiple comparisons |
| ☐ | ☒ | A full description of the statistical parameters including central tendency (e.g. means) or other basic estimates (e.g. regression coefficient) AND variation (e.g. standard deviation) or associated estimates of uncertainty (e.g. confidence intervals) |
| ☒ | ☐ | For null hypothesis testing, the test statistic (e.g. $F$, $t$, $r$) with confidence intervals, effect sizes, degrees of freedom and $P$ value noted<br>*Give P values as exact values whenever suitable.* |
| ☒ | ☐ | For Bayesian analysis, information on the choice of priors and Markov chain Monte Carlo settings |
| ☒ | ☐ | For hierarchical and complex designs, identification of the appropriate level for tests and full reporting of outcomes |
| ☒ | ☐ | Estimates of effect sizes (e.g. Cohen's $d$, Pearson's $r$), indicating how they were calculated |

*Our web collection on statistics for biologists contains articles on many of the points above.*

## Software and code

Policy information about availability of computer code

| | |
|---|---|
| Data collection | The manuscript does not collect empirical data. |
| Data analysis | All the codes associated with the numerical simulations and analysis of this manuscript are available at the Github repository (https://github.com/twang1993/DiversityByGeneTransfer). |

For manuscripts utilizing custom algorithms or software that are central to the research but not yet described in published literature, software must be made available to editors and reviewers. We strongly encourage code deposition in a community repository (e.g. GitHub). See the Nature Portfolio guidelines for submitting code & software for further information.

## Data

Policy information about availability of data

All manuscripts must include a data availability statement. This statement should provide the following information, where applicable:

- Accession codes, unique identifiers, or web links for publicly available datasets
- A description of any restrictions on data availability
- For clinical datasets or third party data, please ensure that the statement adheres to our policy

The simulation data generated in this study have been deposited in the Github repository (https://github.com/twang1993/DiversityByGeneTransfer). Source data are provided with this paper.

## Research involving human participants, their data, or biological material

Policy information about studies with human participants or human data. See also policy information about sex, gender (identity/presentation), and sexual orientation and race, ethnicity and racism.

| | |
|---|---|
| Reporting on sex and gender | NA |
| Reporting on race, ethnicity, or other socially relevant groupings | NA |
| Population characteristics | NA |
| Recruitment | NA |
| Ethics oversight | NA |

Note that full information on the approval of the study protocol must also be provided in the manuscript.

# Field-specific reporting

Please select the one below that is the best fit for your research. If you are not sure, read the appropriate sections before making your selection.

☐ Life sciences ☐ Behavioural & social sciences ☒ Ecological, evolutionary & environmental sciences

For a reference copy of the document with all sections, see nature.com/documents/nr-reporting-summary-flat.pdf

# Ecological, evolutionary & environmental sciences study design

All studies must disclose on these points even when the disclosure is negative.

| | |
|---|---|
| Study description | This study is purely theoretical and does not collect any empirical data. We developed a theoretical framework to analyze how horizontal gene transfer influences the diversity and coexistence of competing microbes. We applied this framework to populations of two or multiple species. In each analysis, we changed the gene transfer rate, and performed numerical simulations with randomized parameters to calculate the consequent coexistence feasibility or species diversity. |
| Research sample | The numerical simulations were carried out by randomly sampling parameters in the given parameter space. The parameter ranges was chosen around the physiological values of different parameters. |
| Sampling strategy | Parameters were randomly sampled from the given space following uniform distribution or normal distribution. Sample sizes are larger than 500 in each simulation. No sample size calculation was performed. The sample sizes in this study is large enough for our analysis. |
| Data collection | No empirical data were collected in this study. All results were generated by numerical simulations. |
| Timing and spatial scale | The timing scale in the simulations is within 200 hours. Since the theoretical framework assumes the populations are well mixed, spatial scale is not relevant in this study. |
| Data exclusions | No data were excluded in this study. |
| Reproducibility | All simulations were repeated at least three times. Each replication led to the same conclusion. |
| Randomization | This is a theoretical study and did not collect empirical data. Randomization is not relevant. |
| Blinding | This is a theoretical study and did not collect empirical data. Blinding is not relevant. |

Did the study involve field work? ☐ Yes ☒ No

# Reporting for specific materials, systems and methods

We require information from authors about some types of materials, experimental systems and methods used in many studies. Here, indicate whether each material, system or method listed is relevant to your study. If you are not sure if a list item applies to your research, read the appropriate section before selecting a response.

## Materials & experimental systems

| n/a | Involved in the study |
| --- | --- |
| ☒ | ☐ Antibodies |
| ☒ | ☐ Eukaryotic cell lines |
| ☒ | ☐ Palaeontology and archaeology |
| ☒ | ☐ Animals and other organisms |
| ☒ | ☐ Clinical data |
| ☒ | ☐ Dual use research of concern |
| ☒ | ☐ Plants |

## Methods

| n/a | Involved in the study |
| --- | --- |
| ☒ | ☐ ChIP-seq |
| ☒ | ☐ Flow cytometry |
| ☒ | ☐ MRI-based neuroimaging |
